# Comparative Analysis of Machine Learning Techniques for Identifying Multiple Force Systems from Accelerometer Measurements

**DOI:** 10.3390/s24206675

**Published:** 2024-10-17

**Authors:** Giovanni de Souza Pinheiro, Fábio Antônio do Nascimento Setúbal, Sérgio de Souza Custódio Filho, Alexandre Luiz Amarante Mesquita, Marcus Vinicius Alves Nunes

**Affiliations:** 1 Institute of Technology, Federal University of Pará, Belém 66075-110, Brazil; fabioans@ufpa.br (F.A.d.N.S.); alexmesq@ufpa.br (A.L.A.M.); mvan@ufpa.br (M.V.A.N.); 2 Marabá Industrial Campus, Federal Institute of Pará, Marabá 68740-970, Brazil; sergio.custodio@ifpa.edu.br

**Keywords:** force identification, vibration measurements, finite element method, harmonic analysis, machine learning

## Abstract

The knowledge of the forces acting on a structure enables, among many other factors, assessments of whether the component’s useful life is compromised by the current machine condition. In many cases, a direct measurement of those forces becomes unfeasible, and an inverse problem must be solved. Among the solutions developed, machine learning techniques have stood out as powerful predictive tools increasingly applied to engineering problem-solving. This study evaluates the ability of different machine learning models to identify parameters of multi-force systems from accelerometer measurements. The models were assessed according to their prediction potential based on correlation coefficient (R^2^), mean relative error (MRE), and processing time. A computational numerical model using the finite element method was generated and validated by vibration measurements performed using accelerometers in the laboratory. A robust database created by the response surface methodology in conjunction with Design of Experiment (DOE) was used for the evaluation of the ability of machine learning models to predict the position, frequency, magnitude, and number of forces acting on a structure. Among the six machine learning models evaluated, k-NN was able to predict with a 0.013% error, and Random Forests showed a maximum error of 0.2%. The innovation of this study lies in the application of the proposed method for identifying parameters of multi-force systems.

## 1. Introduction

The knowledge of parameters such as location, frequency, and magnitude of dynamic forces that excite a system is highly important in virtually all engineering disciplines, including civil, mechanical, aerospace, and electrical engineering. Examples involve the identification of impacts on civil structures [1], stresses in wind turbine blades [2,3], and identification of force transmission in transformer cores and power reactors [4,5].

Knowing those parameters is important for a structural dynamics analysis, as (“pois”) the knowledge of the dynamic loads acting on a structure enables evaluations of stresses to which the system components are exposed. Since the efforts tend to be harmonic and produce cyclic deformations, a particularly important parameter to be considered is fatigue failure, which is one of the main reasons for component failures under cyclic excitations despite those loads being lower than the static resistance of the material.

The knowledge of location, magnitude, and frequency of excitation of forces acting on a structure promotes a detailed evaluation of stresses and deformations undergone by the components over time. Such an analysis is crucial for predictions of the lifespan of materials, thus preventing catastrophic failures and optimizing preventive maintenance. Moreover, the identification of acting forces helps improve engineering designs, resulting in more robust and efficient equipment. Advanced techniques for the detection and analysis of those forces are indispensable for the continuity and safety of high-voltage systems in various industrial and infrastructure applications [6,7,8].

However, determining the location and magnitude of dynamic forces that excite a system is not always simple, especially when the operational forces cannot be directly measured, which occurs in inaccessible locations. Consequently, transducers cannot be introduced into the structure, limiting the analysis to a few available positions [9].

In structures such as wind turbines or aircraft wings, the knowledge of external loads is fundamental, since a direct measurement of the forces applied to the structures is often impossible. A solution is the use of the inverse force identification problem, in which the determination of the number of forces applied and their location of application and magnitudes is desired.

Many of those problems are solved by indirect measurement techniques (inverse problems), whose approach consists in computing the input parameters of a system (excitation loads) through known output data (vibration) and the structure model. They can also be solved by methods such as pseudo-inversion for over-determined systems, Kalman filters, Singular Value Decomposition, and Tikhonov regularization [10].

According to Rezayat et al. [1], the major challenge of inverse problems is they are mathematically ill-posed in almost all cases—i.e., there is not a unique solution to the problem that continuously depends on the measured data. Such a lack of stability is related to the fact that the system model is deficient in classification, and the condition number is high. In other words, a wide range of unrealistic but mathematically correct solutions may exist, resulting in the same structural response.

Kim and Nelson [11] claimed that state force locations are often known and can be approximated as point forces. Therefore, indirect methods can be adopted—a matrix of measured frequency response functions (FRFs) is inverted and used to reconstruct the operational force spectra from a set of measured operational responses in the frequency domain. The accuracy of source strength reconstruction in the presence of noise that contaminates the measured pressures is crucially dependent on the conditioning of the matrix to be inverted. In such situations, small errors in the operational responses can lead to large errors in the reconstructed forces.

In practical engineering, both the location and reconstruction of external excitations pose a challenge due to the large dimension of the problem and the lack of prior information on the accuracy and shape parameters of the forces [2].

In general, load identification problems are treated differently in the function of the choice of the working domain. Studies aimed at force identification by the inverse method have faced great difficulty in the temporal representativeness of the measurements so that the outputs can be known at several measurement points while the system is excited [12].

Machine learning has been explored for pattern identification in high-dimensional data, and machine learning models consist of algorithms through which a computer can learn from empirical data, modeling linear or non-linear relationships between the properties of materials and related factors [13]. According to Yang et al. [14], a model learning the training set shows a better prediction effect on the test set.

Buonanno et al. [15] emphasized that machine learning models can refine the prediction of a prediction model, even in a regime with few data, and Barbaresi et al. [16] claimed that computational time can be significantly reduced through the application of machine learning models to bypass energy simulations.

Setúbal et al. [12] identified parameters of a force in a plate using a model in the finite element method (FEM) validated by experimental vibration measurements. A database based on the design of experiment (DOE) and response surface method (RSM) was created, and the Random Forests Method identified the system parameters, yielding excellent results related to the identification of systems with a force.

The response surface methodology (RSM) is an optimization method that combines the response surface of an experimental sample dataset, provides the surface equation, and then solves the surface equation to obtain a set of optimal design variables [17].

Unlike other statistical methods, RSM not only considers the interaction between independent variables and improves the accuracy of the fit but also uses graphical technology to display the functional relationship between the two, making the results more intuitive [18].

This study is an extension of an investigation conducted by Setúbal et al. [12] towards identifying the method’s capability to handle multi-force systems and other machine learning models that accurately predict such types of problems. It evaluates the ability of different machine learning models to identify multi-force systems, which is an aspect not yet explored in the literature. Six machine learning models were compared regarding prediction capability for parameters such as position, magnitude, number of forces, and frequency of the forces from accelerations measured at each point. The models were selected in function of their ability to handle nonlinear data.

The methodological procedure consisted firstly of an experimental modal analysis conducted on a steel plate involving 49 acceleration measurement points. A computational model in the finite element method (FEM) was generated and calibrated by the experimental vibration results measured and validated according to the Modal Assurance Criterion (MAC) and Coordinate Modal Assurance Criterion (COMAC) [19,20,21]. A harmonic analysis was then conducted with the help of DOE applied to the force parameters of the model based on Central Composite Design (CCD) and varying both frequency and magnitude of the forces. The results were extrapolated with the use of RSM, which made the database much more robust and thus used for evaluations and comparisons of the prediction capability of the following six machine learning models: Random Forest Method (RFM) [22], k-nearest neighbors Method (k-NN) [23,24], Random Tree Method (RTM) [25], XGBoost Tree Method (XGBTM) [26], AS Tree Method (ASTM) [27], and Neural Network Multilayer Perceptron Regressor (NNMLP) [28,29]. This study was conducted first considering the forces at the 49 points used in the experimental procedure. However, the possibility of reducing the number of force application points without decreasing the efficiency of the methods can be evaluated through an analysis of feature relevance.

## 2. Methodology

**Figure 1:**
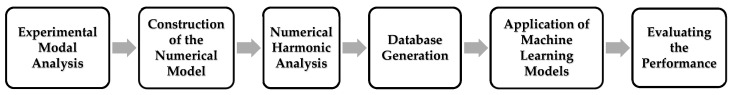
Flowchart of the fundamental steps taken for achieving the study objective.

This study proposes solving the identification of multi-force systems through the resolution of an inverse problem, in which the forces acting on the system are obtained from vibration data. A flowchart was created to facilitate the understanding of the methodological process (see Figure 1). It displays the methodology used for the identification of the excitation forces to the detriment of the vibration responses generated by a computational numerical model in FEM.

First, an experimental modal analysis was conducted in the laboratory for determining the natural frequencies and associated modal shapes of the structure. A computational model was generated from the experimental model, calibrated by the experimental results, and then validated by MAC and COMAC.

A harmonic analysis of the numerical model in FEM was performed with the application of two forces to the structure, involving 49 points of force and measurement of their average acceleration at each new position of force. DOE and RSM were used for the creation of the database. The procedure made the database much more robust, which was thus used for evaluations of the following six regression models: Random Forest Model, k-NN Model, AS Tree Model, Random Tree Model, XGBoost Tree Model, and Neural Network MLP Model.

The performance of each machine learning method was assessed through the coefficient of determination (R^2^), mean relative error, and processing time. Each step is displayed in the flowchart of Figure 1 and detailed in the following sections.

### 2.1. Experimental Modal Analysis

Modal analysis is a process through which a structure can be described in terms of its natural characteristics, namely natural frequencies, damping factors, and modal shapes—i.e., its dynamic properties [30]. Therefore, the system must be excited, its response must be acquired, and the data must be processed, so that the properties can be obtained through methods of extracting modal parameters.

The modal parameter extraction method was applied in the frequency domain according to measured response functions, with measurements of both the force applied to the system by a load cell applied to the vibration exciter and its response using an accelerometer piezoelectric scanning the structure. With excellent dynamic characteristics, piezoelectric devices enable detecting periodic forces with wide frequency ranges and rapid changes caused by impact forces [31].

**Figure 2:**
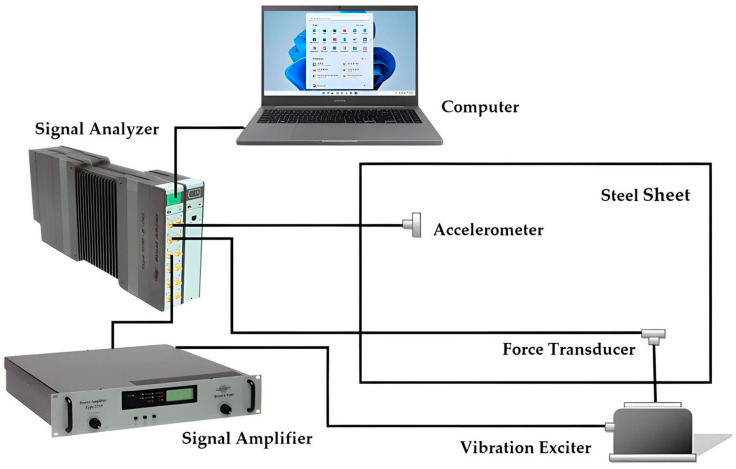
Basic measurement system used in experimental modal analysis with vibration exciter (Shaker), force transducer, and accelerometer.

Figure 2 displays a basic diagram of the measurement system used that generates vibrations and captures force and response signals. The experimental setup can be subdivided into two branches, of which one induces system vibration (internal signal generator of the microcomputer board, power amplifier, and vibration exciter) and the other enables measurement and analysis of force and response signals (transducers, pre-amplifiers if applicable, and analyzer acquisition board). 

The measurement system consisted of an accelerometer (PCB 353B16), a force sensor-type load cell (B&K 8001), a vibration exciter (B&K 4809), a signal amplifier (B&K 2716), a signal analyzer (B&K 3160-B-042) with Pulse Labshop software version 16.1.0, and a notebook (see Figure 2). The accelerometer used was manufactured by PCB Piezotronics (Depew, New York, NY, USA) and the other devices used are manufactured by Brüel & Kjær Sound & Vibration Measurement A/S (Nærum, Denmark).

A 3 mm thick 420 mm × 360 mm steel sheet was suspended by flexible nylon threads for the test. Due to the highly flexible suspension, the suspension/rigid body assembly shows very low natural frequencies associated with rigid body modes, albeit still far from the natural frequencies corresponding to actual vibration modes. The structure can be approximated as a simple pendulum for such a pendulum-like configuration.

**Figure 3:**
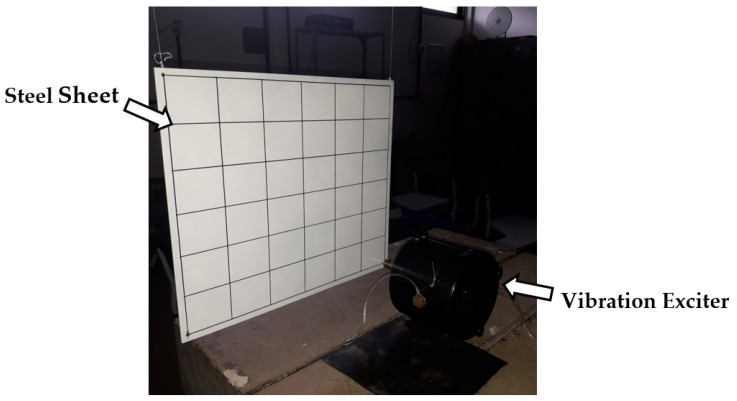
Experimental system: suspended sheet with vibration exciter at point 49.

A measurement mesh with a 10 mm offset from the edges was drawn on the sheet, covering 49 measurement points. Each point was 66.2 mm horizontally and 56.7 mm vertically distanced from the other, therefore forming a mesh with seven lines and seven columns. The excitation force was fixedly positioned at point 49, as this location can excite a greater number of sheet modes. Measurements were taken by the accelerometer at each intersection of horizontal and vertical lines at locations 1 to 49. The sheet with the vibration exciter at point 49 is displayed in Figure 3.

The 49 points were established on the front part of the sheet, starting from top to bottom and from left to right (see Figure 3), selected for measuring vibration. Both excitation and responses are perpendicular to the surface of the sheet. An experimental modal analysis determined the values of the natural frequencies of the system, which were used for validating the computational numerical model, which will be described in greater detail.

### 2.2. Computational Numerical Model Using Finite Element Analysis

The numerical computational model was constructed from the results of the experimental modal analysis, the plate data and parameters, as well as the results, and validated with the values of natural frequencies and associated modal shapes, according to the Modal Assurance Criterion (MAC) and Coordinate Modal criteria. Assurance Criterion (COMAC) was applied for the generation of the database.

Table 1 shows the values adopted for the sheet properties after model calibration.

The finite element of the structural shell element type of four nodes (SHELL181) was used for the creation of the finite element model of the plate, since it fitted very well with the characteristics of the evaluated system. SHELL181 is suitable for analyses of thin to moderately thick shell structures and has four nodes with six degrees of freedom each: translations in x, y, and z directions and rotations around x, y, and z axes.

**Figure 4:**
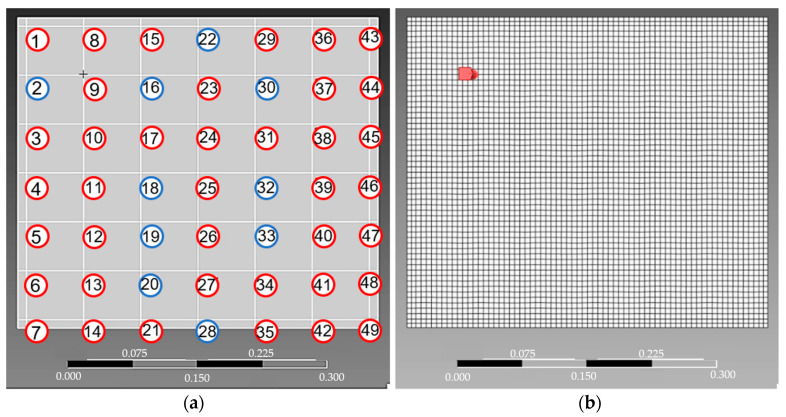
Computational geometry: (**a**) measurement point locations; (**b**) finite element mesh.

Figure 4a illustrates the geometry of the model with details of the discretization used, corresponding to the measurement mesh applied experimentally on the plate. A structured mesh containing 4060 elements and 4129 nodes was achieved through a mesh convergence analysis (Figure 4b).

The points highlighted in blue in Figure 4a correspond to the most important measurements and will be discussed in the Section 3.

### 2.3. Numerical Harmonic Analysis Using Finite Element Analysis

**Figure 5:**
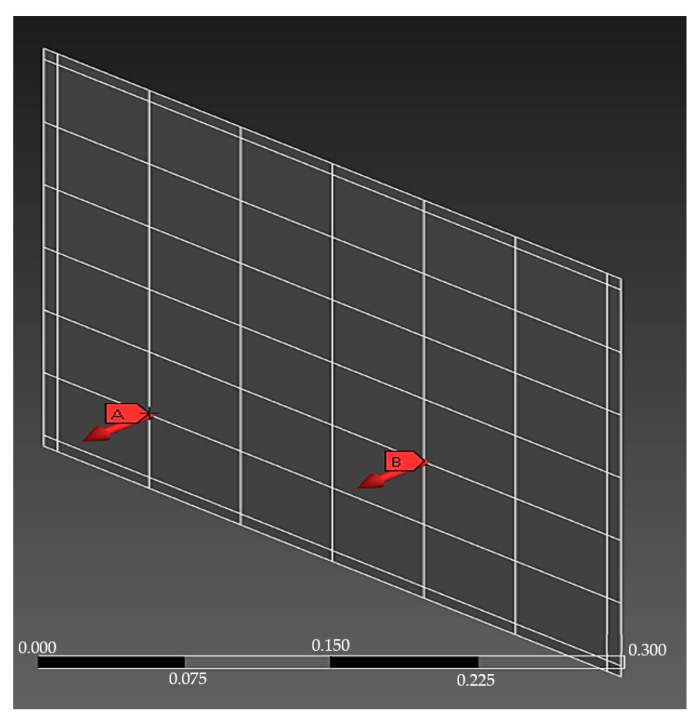
Numerical harmonic analysis with fixed force positioned at point 33 and variable force positioned at point 13.

Harmonic analysis determines the steady-state response of the system due to the application of a harmonic load of known frequency and amplitude. Two conditions for the test were used. In the first, only one force swept the entire structure (49 points), whereas in the second, two forces were used for the numerical harmonic analysis, of which one remained fixed at point 33 and the other varied in position with each new analysis (49 points). The forces were applied perpendicularly to the model plane (Z direction)—the position of only one of the forces was changed after each data acquisition for covering the entire measurement grid (49 points), whose creation was based on the experimental model. Figure 5 shows the force fixations at points 13 and 33.

### 2.4. Database Generation

A harmonic analysis was performed for each force position, and 49 vibration results were acquired for each condition, totaling 4802. The design of experiments (DOE) was used to vary the magnitude of the variable force from 1 mN to 5 mN and frequency from 0 Hz to 250 Hz at each point of the numerical harmonic analysis, whereas the magnitude of the fixed force was fixed at 5 mN.

DOE was configured with the Central Composite Design (CCD) design type and self-defined design, which optimizes the design according to the number of input parameters.

The results were compiled into a single file containing 245,000 lines of data and 53 columns (variable force position, variable force frequency, variable force magnitude, fixed force magnitude, and 49 vibration responses).

### 2.5. Models in Machine Learning

Machine learning is a method that enables computer systems to automatically improve and optimize performance by learning patterns and rules from data. It discovers patterns and makes predictions or data-driven decisions by building and training models [32].

According to Tucci [33], the success of machine learning depends on both the quantity and quality of available data. Accurate well-labeled data can significantly improve the effectiveness of machine learning algorithms and the choice of the right algorithm for the right problem is crucial, since each algorithm has its own strengths and limitations. Therefore, a deep understanding of the problem and the available data is essential.

The dataset was initially randomly separated for training the regression models—80% were used for training and 20% were employed for testing the trained model, following the methodology presented by Breiman and Setúbal [12,22].

Six machine learning models, namely Random Forest Model, k-NN Model, AS Tree Model, Random Tree Model, XGBoost Tree Model, and Neural Network MLP Model, were considered in function of their prediction performances.

Parameters R^2^ and mean relative error were considered for evaluations of the models.

### 2.6. Evaluation of the Performance of Machine Learning Models

Statistical metrics were adopted as a model evaluation procedure. The coefficient of determination (R^2^), according to Equation (1), is used as a measure of the amount of variation in real data represented by the model.

(1) $$R^{2}=1-\frac{{\sum_{i=1}^{n}\left(X_{i}-Y_{i}\right)}^{2}}{{\sum_{i=1}^{n}\left(Y-Y_{i}\right)}^{2}}$$

Another metric is mean relative error (MRE), described in Equation (2):

(2) $$MRE=\frac{1}{n}\sum_{i=1}^{n}\left|\frac{x_{i}-y_{i}}{x_{i}}\right|$$

The calculated metrics were used for comparisons of the models in terms of predictive capability. The execution time of the “running code” (another metric adopted) was measured for all models for the determination of modeling speed.

## 3. Results

### 3.1. Results of Experimental and Numerical Modal Analyses

An experimental modal analysis aims to identify the resonant frequencies of the structure using the FRF-type signal, which can be performed through the analysis of the Bode diagram where vibration amplitudes and their respective phase angles are shown. A resonant frequency can be identified when a peak in vibration amplitude appears associated with a ±90° phase angle. Modal parameter extraction methods also enable the visualization of modal shapes and modal damping of the structure.

**Figure 6:**
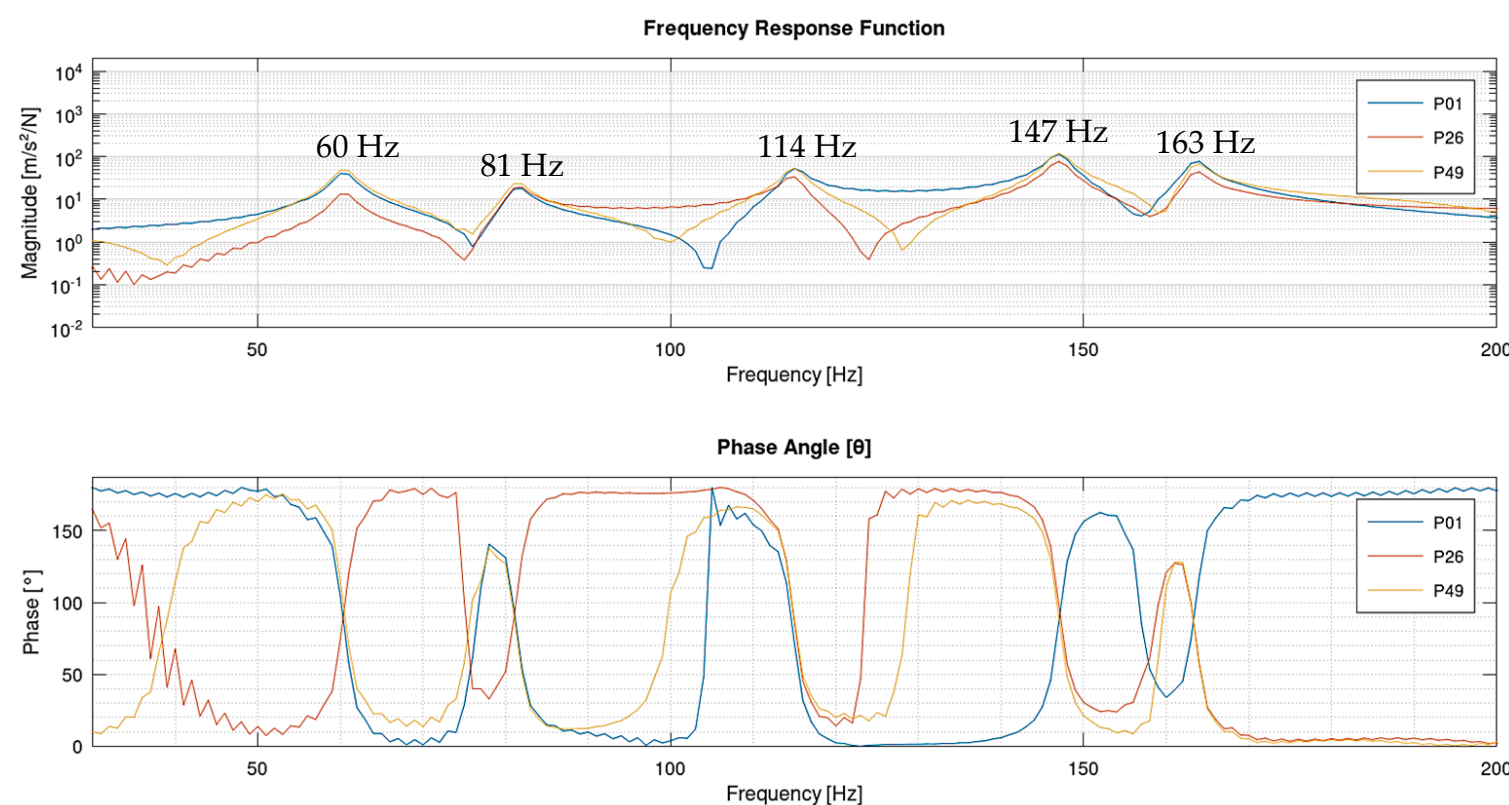
Frequency response function of points 01, 26, and 49 on the plate highlighting the first five natural frequencies.

Figure 6 displays the Bode diagram of the point measurement of the inertance type measured at point 01 (P01), point 26 (P26), and point 49 (P49), characterized by its anti-resonances (decreasing peaks). At least five resonant frequencies, namely, 60 Hz, 81 Hz, 114 Hz, 147 Hz, and 163 Hz, can be identified near the frequencies, as shown by the associated phase angles of ±90°.

**Figure 7:**
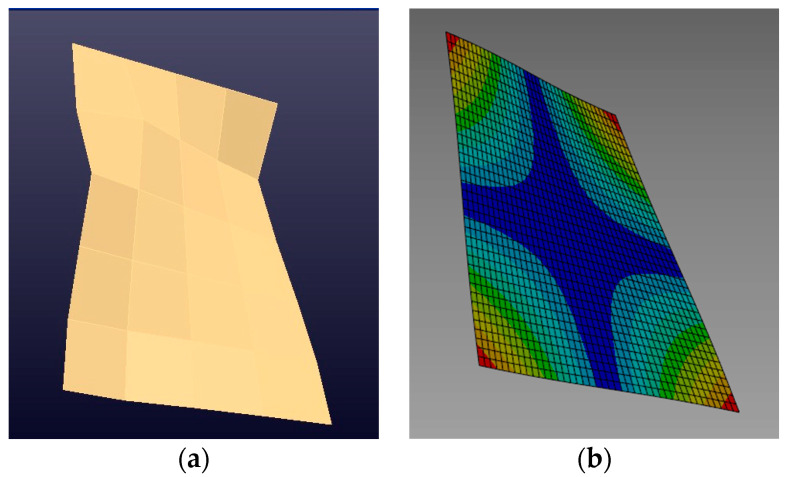
Mode shape associated with the first bending mode, 60 Hz: (**a**) experimental model (**b**) numerical model.

Figure 7a shows the vibration mode shape of the plate associated with its first natural frequency. The image was generated through FRFs measured during the experimental modal analysis. Figure 7b displays the vibration mode shape of the plate associated with its first natural frequency generated from the calibrated numerical FE model. The equivalence in the mode shape in both images for the natural frequency presented, approximately 60 Hz, can be observed.

**Figure 8:**
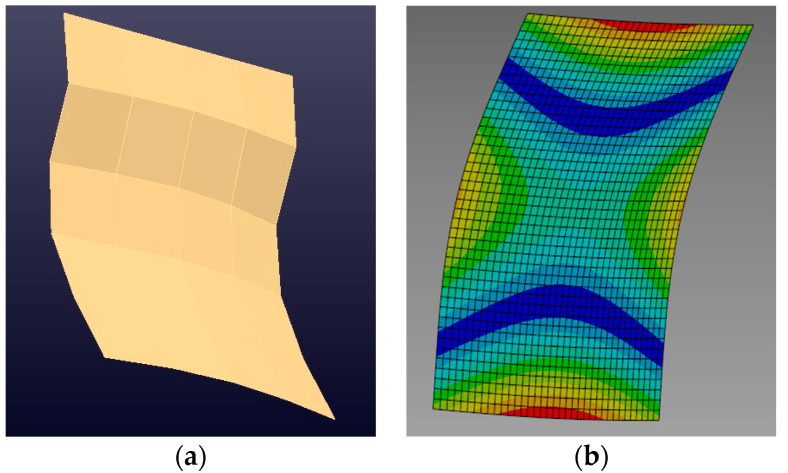
Modal shape associated with the second bending mode, 81 Hz: (**a**) experimental model (**b**) numerical model.

Figure 8 shows the second bending mode with an 81.4845 Hz natural frequency obtained by processing the experimental results. The value is close to the natural frequency of 81.369 Hz, generated from the calibrated numerical FE model.

In Figure 7b and Figure 8b, the points with the highest displacement amplitude are shown in warm colors, tending towards red, while those with the lowest amplitude are shown in cool colors, tending towards blue.

**Figure 9:**
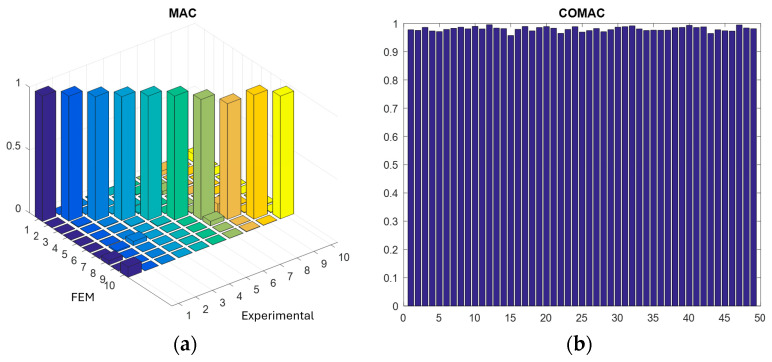
Comparison of natural frequencies and modal forms of experimental and numerical models: (**a**) graph with MAC values; (**b**) graph with COMAC values.

Natural frequencies and modal shapes were compared by MAC and COMAC, considering the first 10 modes obtained experimentally, and through the calibrated FEM computational model. When two vectors establish a linear relationship, the MAC value tends to be close to one. Therefore, according to the MAC (Figure 9a), the values of the diagonal modes, which relate the values obtained from the experimental modal analysis of the plate and the values obtained from the computational modal analysis via FEM, were close to one, showing a strong correlation between the models studied. Figure 9b shows that the COMAC also provided results close to one, which highlights the good correlation between the model’s degrees of freedom.

### 3.2. Database Generation

Harmonic analyses were conducted after the validation of the computational model. A force was inserted at each measurement point, perpendicular to the model plane (Z direction). The position was changed after each data acquisition so that the entire measurement mesh (49 points) could be covered. A second force was fixed at point 33, with a 5 mN magnitude, remaining fixed at that point at each position of the mobile force. The force positioning points and responses were defined according to the experimental model and are described in greater detail in the Methodology chapter.

**Figure 10:**
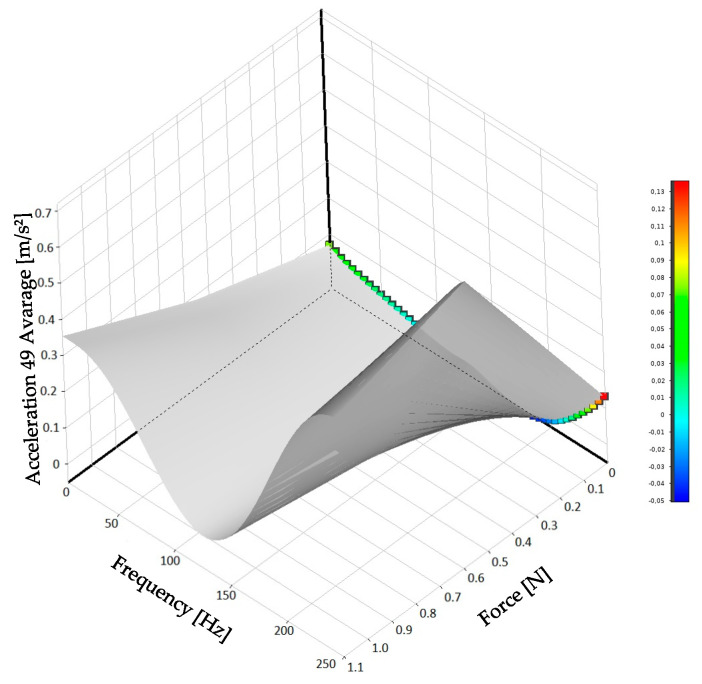
Response surface for acceleration at point 49 as a function of the force component at point 33 and frequency.

DOE, in conjunction with the response surface method, was used for expanding the database. Figure 10 shows the response surface generated for the model with the moving force at point 33 and response at point 49.

According to Figure 10, there is no linearity among the studied variables. The knowledge of such a relationship is important, as it enables the understanding of how a selected output parameter changes as a function of variations in input parameters. Therefore, a nonlinear machine learning model should better fit the dataset.

**Figure 11:**
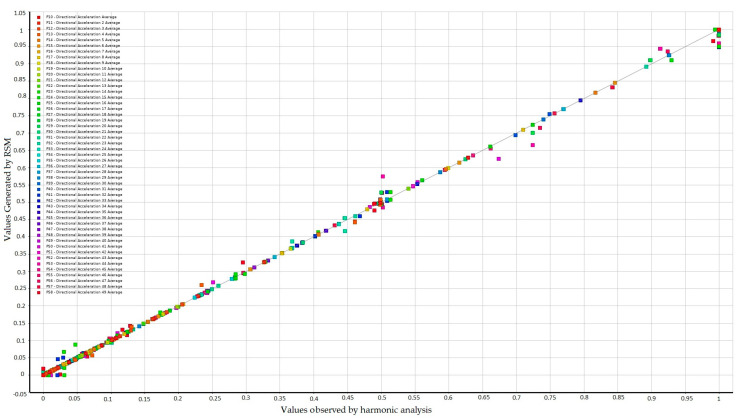
Comparison between the predicted response surface values and the points generated by the harmonic analysis for the force at point 40.

Figure 11 displays a comparison between the predicted response surface values and the points generated by the harmonic analysis for the force at point 40. The data tend to fit linearly into a line at 45°, showing a strong consistency between the data generated by the harmonic analysis and those extrapolated by the RSM.

The procedure generated 49 response surfaces for each position of the moving force (49 positions), totaling 2401 response surfaces. As a result, the database generated by the RSM has 245,000 rows and 53 columns.

### 3.3. Building Models in Machine Learning

**Figure 12:**
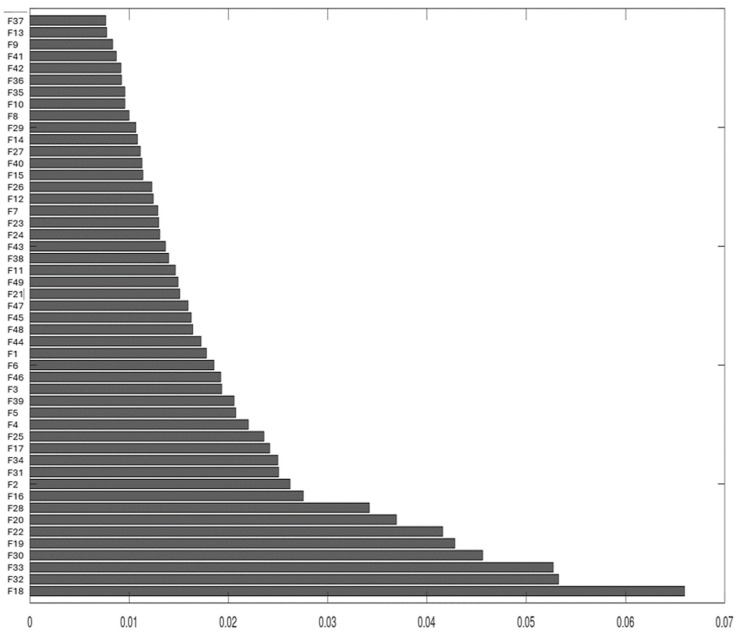
Importance of vibration measurement locations (features).

An analysis of the degree of importance of each feature (average accelerations at each point) in the prediction of machine learning models was initially conducted for investigations on “redundancies” in the data (number of features). Features are represented by F, followed by their respective measurement point, as shown in Figure 12.

According to the percentage importance graph for the model (Figure 12), feature 37 (F37), referring to measurement point 37, as well as other features offer a low contribution to the construction of the models (below 1%). Such information is important for the definition of a more appropriate number of features for the construction of the models.

Six machine learning models, namely Random Forest, k-NN Algorithm (k-Nearest Neighbor), Random Trees, AS Tree, XGBoost Tree, and neural network MLP, were used. Regression models were adopted because the data consist of continuous variables.

The dataset was initially randomly separated for training the regression models—80% were used for training, and 20% were employed for testing the trained model. The models were compared according to R^2^ factor, mean relative error, and processing time.

Table 2 shows the results of the machine learning models.

According to Table 2, the six machine learning models showed a good correlation with the experimental model. However, Random Forest and k-NN best adjusted to force prediction—Random Forest found errors below 1%, and k-NN predicted data with 0.013% error.

Regarding processing times, the models provided similar results—k-NN showed the best times. k-NN did not produce results for the model with 49 features, as it extrapolated the maximum grouping settings, and processing time was configured so as to not make the model too robust.

According to the reduction in the number of features shown in Table 2, k-NN and Random Forest maintained their predictive power, despite the reduction to 10. In other words, with only 10 measurements in the super surface of the sheet, the proposed model can identify the number of forces acting on the structure, in addition to their frequencies, magnitudes, and locations. The ten points adopted are highlighted in blue in Figure 4a, and their importance is displayed in Figure 12.

Table 3 shows the accuracy level of k-NN and Random Forest for 10 features, considering training and testing stages.

Table 3 shows that 196,000 features were used for training, and 49,0000 were used for testing the models. k-NN failed to predict the results 42 times in training and 6 times in testing, whereas Random Forest failed to predict results 196 times during training and 99 times during testing, demonstrating the great potential of the methodology.

## 4. Conclusions

An analysis of the academic literature revealed many researchers have directed their energies towards the development of sophisticated numerical and analytical models. Such models aim at identifying and simulating the forces that act on a wide range of systems for optimizing design conditions and minimizing failures and defects when the systems are in operation. The obtaining of accurate information on the dynamic forces that influence these vibratory systems is mandatory and especially relevant for design or diagnostic purposes, as changes in dynamic efforts are directly reflected in the useful life of components (fatigue failure).

Since the articles studied require great experimental and mathematical efforts to reproduce and locate the force in structures by the inverse method, the methodology proposed aims to reduce both time and complexity in the force identification process active in a structure and overcome problems of poor conditioning, normally found in traditional methods for solving inverse problems.

This study identified multi-force systems using the inverse method based on vibration data generated in the laboratory, and the results supported the creation of a computational numerical model in FEM, validated by the MAC and COMAC. Together with the DOE and RSM, a harmonic analysis was conducted and generated a robust database, which was used for evaluating the ability of six machine learning models, assessed according to R^2^ and MRE parameters, to identify multi-force systems.

The machine learning models adopted showed excellent accuracy in predicting force parameters, namely position, magnitude, frequency, and number of forces. k-NN predicted them with errors close to 0%, and Random Forest showed a maximum error of 0.2%. The models revealed high potential to identify multi-force systems.

This study also evaluated the efficiency of the machine learning models by reducing the number of measurement points considered. The models proved capable of providing excellent results with 10 measurement points. The AS Tree, Random Tree, XGBoost Tree, and Neural Network MLP models showed accuracy above 86% considering the use of 49 features. However, the reduction of the number of features to 10 directly impacted the accuracy of these models. This reduction in accuracy is linked to the relevance of each feature in the predictive power of these models. For the case under study, the success in the use of these models is linked to the use of more features, which results in longer experimental procedures.

The results obtained demonstrate the potential of the methodology developed for the identification of multi-force systems, in addition to allowing the determination of the minimum number of crucial points to be measured in the structure without losing efficiency in the identification of the acting forces. Such an analysis is important, as it makes the measurement process much more accurate, directly reflecting in the measurement processes and the time taken during both experimental procedures and data processing.

## Figures and Tables

**Table 1:** Sheet material properties used in numerical models.

Sheet Material Properties	
Young’s Modulus	200 GPa
Specific mass	8135 kg/m^3^
Poisson’s ratio	0.3

**Table 2:** Comparison of prediction metrics for the machine learning models adopted.

Adopted Model	N° of Features	R^2^	MRE [%]	N° of Features	R^2^	MRE [%]	N° of Features	R^2^	MRE [%]
Random Forest	49	1.0	0.1	22	0.998	0.1	10	0.998	0.2
Processing Time	1 min 22 s			1 min 08 s			59 s		
k-NN	-	-	-	22	1.0	0.012	10	1.0	0.013
Processing Time	-			1 min 02 s			56 s		
AS Tree	49	0.904	9.6	22	0.887	11.2	10	0.832	16.9
Processing Time	1 min 25 s			1 min 17 s			1 min 07 s		
Random Tree	49	0.893	11.7	22	0.861	15.6	10	0.805	21.5
Processing Time	1 min 27 s			1 min 18 s			1 min 09 s		
XGBoost Tree	49	0.882	13.7	22	0.830	19.8	10	0.759	27.2
Processing Time	1 min 32 s			1 min 23 s			1 min 12 s		
Neural Network MLP	49	0.863	13.6	22	0.629	37.1	10	0.433	56.8
Processing Time	1 min 27 s			1 min 22 s			1 min 18 s		

**Table 3:** Comparison of prediction errors for k-NN and Random Forest in training and testing phases with the adoption of 10 features.

	k-NN				Random Forest			
	Training	[%]	Test	[%]	Training	[%]	Test	[%]
Hits	195,958	99.992	48,994	99.987	195,804	99.9	48,902	99.8
Error	42	0.008	6	0.013	196	0.1	99	0.2
Total	196,000	100	49,000	100	196,000	100	49,000	100

## Data Availability

The data are available from the corresponding author on reasonable request.
